# Adjustments of cardiac mitochondrial phenotype in a warmer thermal habitat is associated with oxidative stress in European perch, *Perca fluviatilis*

**DOI:** 10.1038/s41598-020-74788-1

**Published:** 2020-10-19

**Authors:** Nicolas Pichaud, Andreas Ekström, Sophie Breton, Fredrik Sundström, Piotr Rowinski, Pierre U. Blier, Erik Sandblom

**Affiliations:** 1 Department of Chemistry and Biochemistry, Université de Moncton, Moncton, NB E1A 3E9 Canada; 2 Department of Biological and Environmental Sciences, University of Gothenburg, 405 30 Gothenburg, Sweden; 3 Department of Biology, Université du Québec à Rimouski, Rimouski, QC G5L 3A1 Canada; 4 Department of Biological Sciences, Université de Montréal, Montréal, QC H2V 2S9 Canada; 5 Department of Ecology and Genetics, Uppsala University, 752 36 Uppsala, Sweden

**Keywords:** Mitochondria, Animal physiology

## Abstract

Mitochondria are playing key roles in setting the thermal limits of fish, but how these organelles participate in selection mechanisms during extreme thermal events associated with climate warming in natural populations is unclear. Here, we investigated the thermal effects on mitochondrial metabolism, oxidative stress, and mitochondrial gene expression in cardiac tissues of European perch (*Perca fluviatilis*) collected from an artificially heated ecosystem, the “Biotest enclosure”, and an adjacent reference area in the Baltic sea with normal temperatures (~ 23 °C and ~ 16 °C, respectively, at the time of capture in summer). Fish were sampled one month after a heat wave that caused the Biotest temperatures to peak at ~ 31.5 °C, causing significant mortality. When assayed at 23 °C, Biotest perch maintained high mitochondrial capacities, while reference perch displayed depressed mitochondrial functions relative to measurements at 16 °C. Moreover, mitochondrial gene expression of *nd4* (mitochondrial subunit of complex I) was higher in Biotest fish, likely explaining the increased respiration rates observed in this population. Nonetheless, cardiac tissue from Biotest perch displayed higher levels of oxidative damage, which may have resulted from their chronically warm habitat, as well as the extreme temperatures encountered during the preceding summer heat wave. We conclude that eurythermal fish such as perch are able to adjust and maintain mitochondrial capacities of highly aerobic organs such as the heart when exposed to a warming environment as predicted with climate change. However, this might come at the expense of exacerbated oxidative stress, potentially threatening performance in nature.

## Introduction

Extreme thermal events (e.g. heat waves) will increase in frequency, duration and intensity around the planet as the climate continues to warm. This may expose animals to physiologically stressful and even lethal temperatures^1–5^. Temperature affects most aspects of the biochemistry and physiology of animals, especially in ectothermic animals as their body temperature is directly determined by the ambient temperature. Thus, it is crucial to understand how ectotherms such as fish respond to gradually increasing temperatures, as well as extreme thermal events to properly understand and manage these ecosystem threats. Heat stress in natural populations can act through different physiological, behavioral and biochemical mechanisms^6–8^. Among these mechanisms, the thermal constraints on aerobic metabolism (the major source of cellular energy, *i.e.* ATP) have been put forward as a limiting factor for animal survival and performance, as the biochemical reactions of this process are profoundly affected, and may even fail at progressively elevated ambient temperatures^8–12^. Yet, the proximate causes of a potential failure to maintain aerobic metabolism in a warming environment remain elusive and highly debated^10,13–15^. This is partly due to the complex integration of processes involved in aerobic ATP production including tissue oxygen supply (*e.g.*, cardioventilatory convective oxygen transport and blood oxygen carrying capacities), as well as mitochondrial capacities and cellular ATP turn-over rates. Nonetheless, highly aerobic organs such as the heart and brain can be predicted to be particularly sensitive to warming, and could represent physiological “bottle-necks” dictating overall organismal thermal sensitivity ^9,10,16,17^.

At the subcellular level, acute warming impacts biochemical reaction rates and catalytic capacities of mitochondrial enzymes. This effect of temperature can be represented by thermal performance curves^10,18,19^. First, the reaction rates increase progressively as temperature increases, followed by a plateau encompassing the thermal optimum (T_opt_). However, extreme temperatures (*i.e.*, typically near an organism’s critical thermal maximum, CT_max_) may cause protein denaturation and misassembly of mitochondrial enzymatic complexes or supercomplexes, as well as increased fluidity of mitochondrial membranes, potentially leading to subsequent loss of structural integrity, impaired kinetic properties and other mitochondrial dysfunctions^10,12,20–24^. This results in sharp decreases of the reaction rates characterized by the steep descending phase of the thermal performance curve. Another potentially negative effect of elevated temperature on cellular homeostasis is the increased generation of reactive oxygen species (ROS) by mitochondria, which causes damage to DNA, lipids and proteins; all being hallmarks of oxidative stress^12,25^. Thus, while mitochondrial respiration may be able to sustain overall ATP demands under moderately elevated temperature, a possible trade-off is ROS overproduction^25–27^. Hence, if the buffering effect of anti-oxidant defenses is not sufficient to prevent the damaging effects of ROS, disturbance of cell homeostasis and possible cell death may occur^12,25,28–30^. Thus, it may then not be surprising that animals exposed to increased habitat temperatures typically exhibit increased levels and/or activities of antioxidant enzymes such as catalase and superoxide dismutase, which neutralize ROS^30–33^. However, if these systems cannot be sufficiently upregulated with chronic and/or acute warming events, oxidative damages may be exacerbated^30,33,34^. Thus, oxidative stress biomarkers are considered good proxies for the metabolic status and health of organisms, and may serve as valuable tools in the management and conservation of wild populations in the context of climate warming^29,30,32,35^.

With prolonged environmental warming, reversible phenotypic plasticity (acclimation) can limit negative impacts of high temperature, and ensure proper regulation of cellular bioenergetics and maintenance of organismal homeostasis^7,36,37^. For example, chronically (several days, weeks or more) warm-acclimated fish generally exhibit metabolic thermal compensation, with decreased rates of mitochondrial oxygen consumption and enzymatic activities^7,10^. Such plastic adjustments can also mitigate warming-induced ROS production^25,26^. Moreover, anthropogenic global warming likely represents a strong selective force that may drive genetic adaptation in natural populations^36^. For example, specific isozymes that can function more optimally at higher temperatures may be selected for^7,38,39^. In addition, mitochondrial DNA (mtDNA) variations have been shown to influence metabolic adjustments to temperature^40–47^. Selection for specific mitochondrial genotypes and/or phenotypes are, therefore, likely to occur with increased average temperatures from climate warming, and could possibly allow natural populations to adapt to warmer thermal habitats^40,41,45,48^. However, a potential trade-off at the cellular level may occur as mitochondrial functions selected for a warmer thermal environment might not be as efficient at transiently low temperatures, which can compromise animal performance in the context of increased temperature variability with climate change^7,38,39^. Even so, knowledge about mitochondrial and cellular responses to acute temperature changes in chronically warm adapted populations in nature is scant.

Here, we assessed the metabolic status of European perch (*Perca fluviatilis*) collected from two thermally distinct ecosystems. One group of perch (Biotest perch) was collected from the “Biotest enclosure”, which is a ~ 1 km^2^ man-made enclosure receiving warm effluent waters from a nuclear power plant, raising the water temperature by ~ 3–10 °C depending on the season, thus representing a chronically warmed ecosystem. The other group of fish (reference perch) was collected from the nearby archipelago where the thermal regime is normal for the region (see ^14^ and ^48^). Previous work has revealed clear differences between Biotest and reference perch regarding cardiac mitochondrial thermal sensitivity and plasticity, differences that remain even after ~ 8 months of acclimation in the laboratory to constant thermal conditions^48^. Importantly, while both populations exhibited higher acute mitochondrial thermal sensitivity when acclimated to laboratory temperatures close to their natural habitat temperatures, this sensitivity was lost when Biotest and reference fish were laboratory-acclimated to opposite temperatures (*i.e.* 16 and 25 °C, respectively, see ^48^). Interestingly, when measured at fixed assay temperatures, ventricular tissue from Biotest fish display generally depressed enzymatic activities, which could serve to prevent accelerated ROS production^49^. They also exhibit changes in cellular membrane composition that could prevent oxidative damage to lipids^49^. While these previous findings may indicate local adaptation of Biotest perch to their warmer environment^48^, it has remained unclear how these populations respond to additional transient heat stress in nature such as during a heat wave.

**Figure 1:**
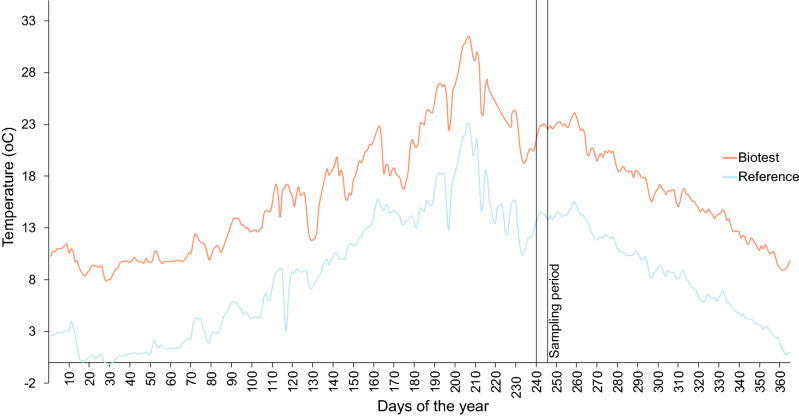
Yearly temperature profiles with daily mean temperatures for the reference area (blue) and the Biotest enclosure (red) in 2014. Fish were collected between the 28^th^ of August and the 3^rd^ of September in 2014. Reference perch were collected from the power plant’s water intake channel (mean temperature at collection time: 14.3 ± 0.2 °C) and Biotest fish were collected from the Biotest enclosure (mean temperature at collection time: 22.8 ± 0.3 °C).

In the present study, we took advantage of an extreme summer heat wave in July 2014 exposing fish to water temperatures peaking at 23.1 °C and 31.5 °C in the reference and Biotest areas, respectively (Fig. 1). By collecting and analyzing fish in late August and early September, this provided an excellent opportunity to examine remaining effects of this preceding heat stress on key mitochondrial functions and oxidative stress parameters across temperatures. Interestingly, some mortalities were reported for the Biotest population during this heat wave (personal communication from Swedish University of Agricultural Sciences), suggesting that surviving perch might have been selected to tolerate warmer temperatures. Thus, we hypothesized that field-sampled perch from the Biotest enclosure would have an improved ability to maintain mitochondrial capacities at elevated temperatures, but that the chronically warm environment should lead to increased oxidative stress due to elevated ROS production. To test these hypotheses, we examined cardiac mitochondrial functions (oxygen consumption rates) of field-collected Biotest and reference perch, approximately one month after the severe summer heat wave (Fig. 1). Both populations were tested at assay temperatures mimicking late summer temperatures of the two locations (16 and 23 °C, respectively). Markers of oxidative damages and activities of antioxidant enzymes were also measured in cardiac tissue of both populations. Finally, we quantified gene expression of mitochondrial complexes I (*nd4*) and IV (*cox1*) subunits and sequenced three mitochondrial genes to evaluate if mitochondrial genomes differed in the two populations, which would provide further evidence of genetic selection to the thermal environments.

## Results

### Temperature profile and morphological variables

Fish were collected between the 28^th^ of August and the 3^rd^ of September in 2014; approximately one month after the highest summer temperatures in the reference area and in the Biotest enclosure were recorded (23.1 °C and 31.5 °C, respectively; see Fig. 1). Reference perch were collected from the power plant’s water intake channel (mean temperature at the time of collection: 14.3 ± 0.2 °C) and Biotest fish were collected from the Biotest enclosure (mean temperature at collection time: 22.8 ± 0.3 °C).

There were no differences between populations in body mass, fork length and condition factor (Table 1). However, the relative ventricular mass of Biotest fish was significantly lower than in reference fish (F_1,38_ = 10.31 with P = 0.003; Table 1) suggesting a structural remodeling of the cardiac tissue.

**Table 1:** Morphological variables of perch (*Perca fluviatilis*) sampled in the reference and the Biotest areas.

Population	M_b_ (g)	FL (mm)	CF	RVM (%)
Reference (N = 20)	415.4 ± 49.9	302.3 ± 9.6	1.40 ± 0.04	0.068 ± 0.002^a^
Biotest (N = 20)	395.5 ± 44.0	293.1 ± 8.2	1.46 ± 0.03	0.062 ± 0.002^b^

M_b_: body mass; FL: fork lenght; CF: condition factor; RVM: relative ventricular mass.

### Cardiac mitochondrial respiration rates and mitochondrial ratios

**Figure 2:**
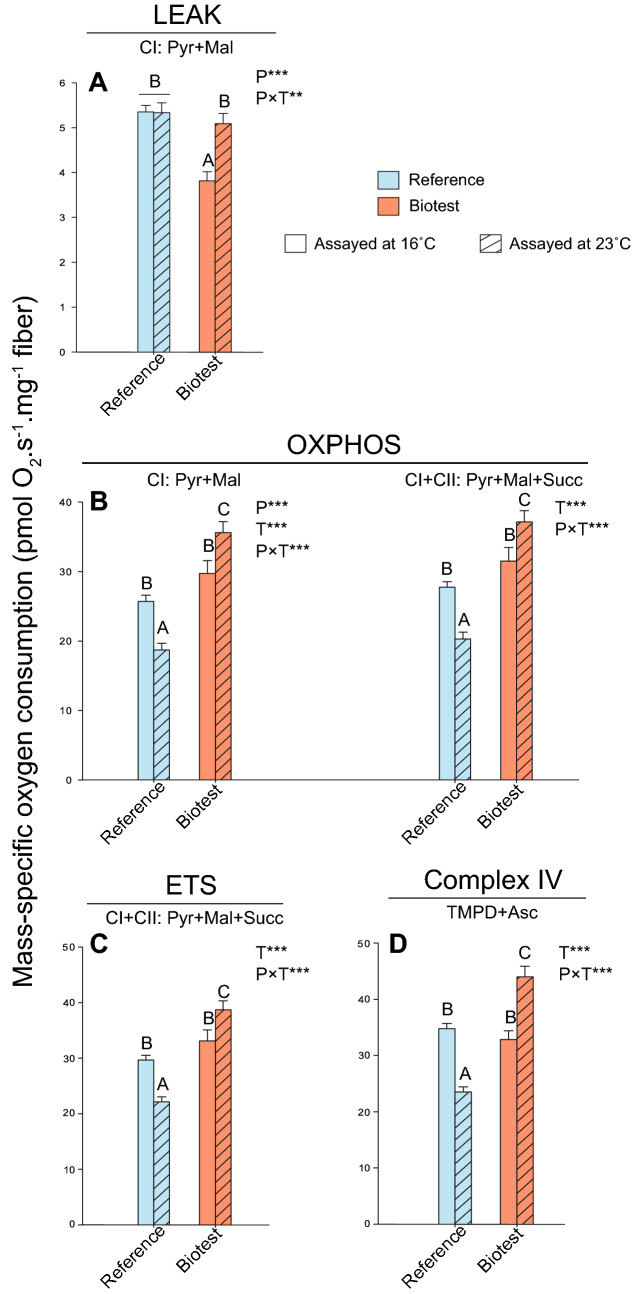
Mitochondrial respiration rates in permeabilized cardiac fibers of perch (*Perca fluviatilis*) collected in the reference and the Biotest areas. Mitochondrial respiration rates were measured during (**A**) the LEAK respiration in presence of pyruvate + malate (CI-LEAK); (**B**) the OXPHOS respiration after addition of ADP (CI-OXPHOS) and succinate (CI + CII-OXPHOS); (**C**) the uncoupled (ETS) respiration after injection of FCCP (CI + CII-ETS); and (**D**) with TMPD + ascorbate (Complex IV) after inhibition of complexes I and III. N = 10 for each population at each assay temperature. Results are means ± s.e.m. Statistical results from two-way ANOVAs are presented for the simple effects as well as for the interaction effect with P: Population effect; T: Assay Temperature effect; P × T: interaction effect; and significance for F-values are represented by *** < 0.001; ** < 0.01; and * < 0.05. Dissimilar letters represent significant differences among groups as tested with pairwise comparisons of the least-squares means using adjusted P-values (Tukey method) with the significance set at P˂0.05.

Cardiac mitochondrial respiration rates were measured at assay temperatures of 16 °C and 23 °C in both Biotest and reference populations (Fig. 2). For each mitochondrial parameter measured, the individual effects of population and assay temperature, as well as the interactions between these factors (population × assay temperature), were evaluated (Fig. 2; Table 2). When assayed at 16 °C, LEAK respiration at the level of complex I (CI–LEAK) was significantly lower in Biotest fish compared to the other groups (Fig. 2A; Table 2; all P-values˂0.001). However, when assayed at 23 °C, CI-LEAK was significantly higher compared to the rates at 16 °C in Biotest fish, while no change between assay temperatures was observed in reference fish. Regarding the other mitochondrial respiration rates, a similar pattern was observed between groups and across assay temperatures *i.e.*: no difference in respiration rates when comparing reference and Biotest perch assayed at 16 °C; increased respiration rates in Biotest fish when assayed at 23 °C compared to the other groups (all P-values˂0.001 for CI-OXPHOS, CI + CII-OXPHOS, CI + CII-ETS and Complex IV; Fig. 2B–D; Table 2); and decreased respiration rates in the reference population when assayed at 23 °C versus 16 °C (P = 0.005, P = 0.003, P = 0.003, and P˂0.001 for CI-OXPHOS, CI + CII-OXPHOS, CI + CII-ETS and Complex IV, respectively; Fig. 2B–D).

**Table 2:** Analyses of variance showing *F* ratios perch (*Perca fluviatilis)* sampled from the reference and Biotest populations.

	Denominator *df*	Population Num *df* = 1	Assay Temperature Num *df* = 1	Interaction Num *df* = 1	Co-variate Weight Num *df* = 1
**Mitochondrial respiration rates**					
CI-LEAK	35	28.34***	0.005	9.98**	0.004
CI-OXPHOS	35	4.24***	13.15***	22.37***	1.05
CI + CII-OXPHOS	35	3.57	14.53***	22.61***	1.56
CI + CII-ETS	35	2.98	14.92***	22.80***	1.15
Complex IV	35	1.23	45.36***	78.92***	4.50*
**Mitochondrial ratios**					
P_I_/L_I_	35	86.45***	12.78**	5.92*	0.80
E_I+II_/P_I+II_	35	10.03**	0.42	2.54	0.50
**Markers of oxidative stress**					
SOD	75	5.90*	1.27	0.08	22.03***
CAT	75	19.56***	0.10	0.11	0.008
TBARS	37	0.006	NA	NA	0.60
Carbonyls	37	108.08***	NA	NA	0.0008
**Relative gene expression**					
*nd4*	17	30.39***	NA	NA	1.65
*cox1*	17	3.99	NA	NA	1.86

* P < 0.05; ** P < 0.01; ***P < 0.001.

**Figure 3:**
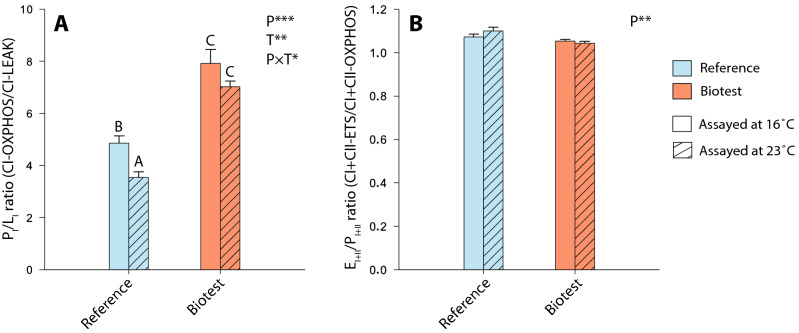
Mitochondrial ratios calculated from respiration rates measured in permeabilized cardiac fibers of perch (*Perca fluviatilis*) collected in the reference and the Biotest areas. (**A**) P_I_/L_I_ ratio was taken as an indicator of mitochondrial integrity and mitochondrial coupling and was calculated as CI-OXPHOS/CI-LEAK; (**B**) E_I+II_/P_I+II_ was determined to estimate the ETS reserve capacity and was calculated as CI + CII-ETS/CI + CII-OXPHOS. N = 10 for each population at each assay temperature. Results are means ± s.e.m. Statistical results from two-way ANOVAs are presented for the simple effects as well as for the interaction effect with P: Population effect; T: Assay Temperature effect; P × T: interaction effect; and significance for F-values are represented by *** < 0.001; ** < 0.01; and * < 0.05. Dissimilar letters represent significant differences among groups as tested with pairwise comparisons of the least-squares means using adjusted P-values (Tukey method) with the significance set at P˂0.05.

The P_I_/L_I_ ratio at the level of complex I (CI-OXPHOS/CI-LEAK), which is an indicator of mitochondrial quality and mitochondrial coupling^21,50^, was significantly higher in the cardiac tissue of Biotest fish compared to reference fish across assay temperatures (P-values < 0.001 for both 16 and 23 °C; Fig. 3A and Table 2). Moreover, in reference fish, this ratio was significantly lower in cardiac tissues when measured at 23 °C compared to 16 °C (P < 0.001), while it remained unchanged across assay temperatures in Biotest fish (Fig. 3A and Table 2). The E_I+II_/P_I+II_ ratio (CI + CII-ETS/CI + CII-OXPHOS) did not differ across populations and assay temperatures (Fig. 3B; Table 2).

### Markers of oxidative stress

Carbonyl content was significantly higher in Biotest compared to reference fish (P˂0.001; Tables 2 and 3), while TBARS levels did not differ between populations. Moreover, activities of anti-oxidant enzymes (SOD and CAT) were consistently higher in Biotest compared to reference perch across assay temperatures, although the interaction population × assay temperature was not statistically significant (Tables 2 and 3).

**Table 3:** Activity of antioxidant enzymes and oxidative damage to lipids and proteins in cardiac tissue of perch (*Perca fluviatilis*) sampled from the reference and Biotest populations.

**Population**	Oxidative damages		Activity of anti-oxidant enzymes			
	TBARS levels (μmol g^−1^ tissue)	Carbonyl content (nmol mg^−1^ proteins)	Superoxide dismutase (U mg^−1^ proteins)		Catalase (mU mg^−1^ proteins)	
			16 °C	23 °C	16 °C	23 °C
**Reference**	0.13 ± 0.02	1.37 ± 0.13***	2.44 ± 0.20	2.77 ± 0.24	9.28 ± 0.65	9.27 ± 0.64
**Biotest**	0.13 ± 0.01	4.62 ± 0.30	3.14 ± 0.28	3.37 ± 0.30	12.62 ± 0.94	13.15 ± 0.96

Fish were sampled in the field where reference and Biotest perch were acclimatized to 16 and 23 °C, respectively. Values are means ± s.e.m. (N = 20). * P < 0.05; ** P < 0.01; *** P < 0.001.

### Mitochondrial gene expression and mtDNA divergence

**Figure 4:**
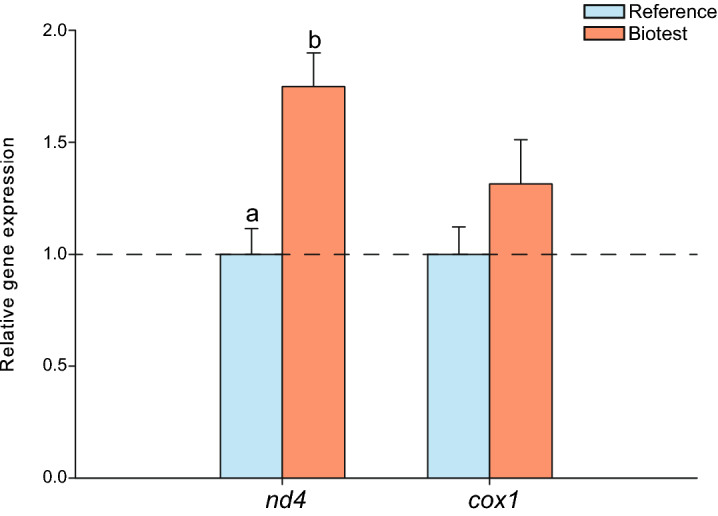
Relative mitochondrial gene expression of *nd4* (complex I subunit) and *cox1* (complex IV subunit) in cardiac tissues of perch (*Perca fluviatilis*) collected in the reference and the Biotest areas. N = 10 for each population. Relative gene expression was calculated with the 2^−ΔΔCt^ method by using the geometric mean of two reference genes (*β-actin* and *ef1-α*). Results are means ± s.e.m calculated with error propagation. Dissimilar letters represent significant differences among groups as tested with pairwise comparisons of the least-squares means using adjusted P-values (Tukey method) with the significance set at P ˂ 0.05.

Biotest fish displayed a significantly higher *nd4* expression than reference fish (P ˂ 0.001), along with a trend for higher *cox1* expression (Fig. 4; Table 2). To determine if these differences were due to mtDNA divergences, we sequenced three different genes; i.e.* 16S*, *cox1* and *cytb* (N = 3 for each population). We only found one single nucleotide polymorphism in *cox1* and one in *cytb* which were both shared between the reference and Biotest populations and did not result in any concurrent changes at the amino–acid level (results not shown).

## Discussion

The present study characterized the thermal effects on cardiac mitochondrial metabolism, oxidative stress, and mitochondrial gene expression patterns in two nearby perch populations from thermally distinct habitats, sampled approximately one month after a severe summer heat wave. Our results show that perch from the Biotest enclosure generally display higher cardiac mitochondrial capacities across assay temperatures, while perch from the reference area displayed depressed mitochondrial functions when assayed at warmer temperatures. In addition, Biotest perch exhibited differences in the expression of mitochondrial genes, presumably contributing to enhance tolerance to environmental warming. Even so, the cardiac tissue from Biotest perch displayed signs of higher oxidative stress despite upregulated antioxidant enzymes. This may reveal a metabolic trade-off in Biotest perch, where improved cardiac mitochondrial capacities at warmer temperature are associated with greater oxidative stress.

Relative ventricular mass was lower in Biotest fish which is consistent with other studies using the same model^14,51^. This remodeling was not surprising and likely constitutes a compensatory mechanism as the reduced ventricular mass in Biotest perch could still sufficiently maintain cardiac output as the force and rate of ventricular contraction typically increase at high temperature^52–54^.

The LEAK respiration rate, which represents mitochondrial oxygen consumption that compensates for proton leak through the inner mitochondrial membrane without ADP phosphorylation, was not different when measured at 16 or 23 °C in reference perch. This indicates that the CI-LEAK should be maximized between 16 and 23 °C in these fish, and that the T_opt_ for this rate lies within this temperature range (Fig. 2A). In Biotest perch, the CI-LEAK was significantly lower than in reference perch when assayed at 16 °C reflecting a change of thermal sensitivity. The other mitochondrial respiration rates (CI-OXPHOS, CI + CII-OXPHOS, CI + CII-ETS, and CIV) were higher in Biotest perch when assayed at 23 °C than when assayed at 16 °C. This result was expected as mitochondrial oxygen consumption increases exponentially with acute increases in temperature due to fundamental thermodynamic effects on molecular movements, explaining the rising phase of thermal performance curves^55–57^. However, in mitochondria of reference fish, reductions in the same rates were observed when measured at 23 °C (Fig. 2), suggesting that this temperature was above T_opt_ for mitochondrial oxygen consumption, and that mitochondrial capacities were in the descending phase of the thermal performance curve. Moreover, the P_I_/L_I_ ratios were much lower in cardiac tissues of reference perch compared to Biotest perch at both assay temperatures (Fig. 3A). This suggests that cardiac mitochondrial dysfunctions occurred in reference fish, especially at warmer assay temperatures, as the P_I_/L_I_ ratio represents the mitochondrial capacity for phosphorylating respiration relative to the respiration required to offset the proton leak, and is usually considered a good indicator of mitochondrial coupling^21,50^. Taken together, our data suggest that the acute thermal performance curve for mitochondrial metabolism has been shifted towards higher temperatures in Biotest perch.

The increased occurrence of oxidative damage to cardiac proteins in Biotest perch suggests that these fish live under more severe oxidative stress, although the TBARS levels were similar between the two populations. We have previously shown that cardiac tissue of Biotest perch is less prone to lipid peroxidation due to changes in cellular membrane composition, which could explain why we did not see a significant increase in TBARS levels even if overall oxidative stress was higher^49^. While a higher capacity of the antioxidant enzyme CAT (and to a lesser extent of SOD) was detected in the Biotest fish, this upregulation did clearly not fully protect this population from the damaging effects of greater oxidative stress in their warmer habitat. As acute heat challenges in fish have been shown to increase ROS production and lead to oxidative damages^58–62^, the increased oxidative stress exhibited by Biotest perch may have resulted from the preceding severe heat stress, as the temperature in the Biotest enclosure approached 31.5 °C approximately one month before the tissue sampling was conducted. In fact, this temperature is close to the whole animal CT_max_ (~ 32 °C) previously determined for this population and should represent a severe heat stress^63^. Interestingly, fish mortality was reported in the Biotest enclosure during the heat wave (personal communication from Swedish University of Agricultural Sciences). Thus, it is possible that the individuals tested in this study represent a thermally selected subset of the Biotest population.

The higher expression of *nd4*, as well as a trend for higher expression of *cox1* mitochondrial genes in Biotest perch may partly explain their better ability to maintain a high respiration rate when assayed at 23 °C compared to reference fish. This could represent a compensatory response allowing the maintenance of mitochondrial functions in a warmer habitat. Indeed, it has been shown in gill tissues of the redband trout (*Oncorhynchus mykiss gairdneri*) from different thermal habitats that an increased expression of mitochondrial genes, especially those encoding complex I subunits such has *nd4*, represents a key molecular adaptation to warmer water temperatures^64^. This increased expression could also be related to mtDNA divergence between the two perch populations examined here. It is possible that mutations and/or selection of available gene variants in mitochondrial or mitochondria–associated nuclear genes have occurred in the population of origin, resulting in changes in gene expression (e.g*. nd4* and *cox1*) and consequently in mitochondrial respiration rates, which is consistent with findings in other species^40,42,45^. However, we did not detect such mitochondrial polymorphisms specific to either population in the three genes examined here. Therefore, without additional data, we cannot currently conclude whether the differences in cardiac mitochondrial functions observed between the reference and Biotest populations can be explained by mtDNA divergences.

In summary, our results show that mitochondrial capacities in Biotest fish have adjusted to tolerate warmer thermal habitats. We cannot exclude the possibility that this phenotype could represent a subset of the Biotest population that had been selected for tolerance to higher temperatures following the preceding summer heat wave. However, this phenotype is also associated with increased oxidative stress. Thus, considering that climate warming is predicted to result in both increased average temperature and more frequent and severe extreme thermal events, our study highlights important consequences and potential threats of future climate warming on fish populations. Moreover, temperature is not the only parameter in our model that could have affected mitochondrial metabolism as other biotic or abiotic factors (or a combination of both) might have participated in the changes observed between the populations. For example, food quality and/or quantity can also modulate mitochondrial structure and function as well as oxidative stress status^65–67^, which in turn could influence the thermal response. While the food availability in the Biotest and in the reference areas, as well as the feeding behavior of perch from the different locations have not been systematically documented^68^, this factor could potentially have influenced our results on mitochondrial metabolism and oxidative stress. The oxygen consumption rate of mitochondria is, however, only one of the functional properties of this organelle, and thermal responses of other functions (regulation of activity, affinity to effectors or substrates, and specific ROS production) could be interesting parameters to investigate in both populations. Our results also suggest that despite depressed mitochondrial respiration rates at higher assay temperatures, reference fish exhibit less pronounced oxidative stress features. The highest assay temperature tested for the reference population (23 °C) was surprisingly almost 7 °C lower than their previously measured CT_max_^63^_._ Thus, future experiments evaluating the relationship between mitochondrial capacities and whole animal thermal tolerance in both populations are required to shed light on the detrimental effects of temperature on longer term animal performance in nature.

## Methods

### Experimental animals and holding conditions

Adult fish of mixed sexes were collected using fishing rods between the 28^th^ of August and the 3^rd^ of September in 2014. Reference perch were collected from the power plant’s water intake channel immediately upstream of the power plant (mean temperature: 14.3 ± 0.2 °C) and Biotest fish were collected from the Biotest enclosure downstream of the power plant (mean temperature: 22.8 ± 0.3 °C), and immediately transported to a nearby wet lab. Thus, both populations were exposed to seawater with essentially the same physical–chemical properties, the only difference being that the water in the Biotest enclosure is warmed when passing the power plant. Moreover, the high water flow (~ 90 m^2^ s^−1^) ensures well oxygenated conditions throughout the ~ 1 km^2^ enclosure (for details see^14^). Fish were held in 1,200 L tanks supplied with a continuous flow of aerated brackish water (~ 5 ppt) from the reference area or the Biotest enclosure for at least 3 days after capture before experiments were performed. The tanks were kept outdoors at a natural photoperiod and the fish were not fed. Fish were netted from the holding tanks and killed with a sharp cranial blow. For all fish, body mass (M_b_) and fork length (FL) were determined. The heart was then quickly excised, and the ventricle was dissected free, blotted and the ventricle mass (M_v_) was determined.

The relative ventricular mass (RVM) was calculated as:

$$ {\text{RVM}} = {\text{M}}_{{\text{v}}} /{\text{M}}_{{\text{b}}} $$

The fish condition factor (CF) was calculated as:

$$ {\text{CF}} = \left( {{1}00{\text{M}}_{{\text{b}}} } \right)/{\text{FL}}^{3} $$

with M_b_ and M_v_ in g and FL in cm.

The ventricle was either directly placed in ice-cold relaxing solution (2.77 mM CaK_2_EGTA, 7.23 mM K_2_EGTA, 5.77 mM Na_2_ATP, 6.56 mM MgCl_2_, 20 mM Taurine, 15 mM Na_2_phosphocreatine, 20 mM imidazole, 50 mM MES,0.5 mM dithiothreitol, pH 7.1) for mitochondrial respiration experiments, or transferred to liquid nitrogen and kept at − 80 °C for further biochemical and molecular assays. Experiments were performed in agreement with the ethical permits 65-2012 and C176/12 from the animal ethics committees in Gothenburg and Uppsala (Sweden), respectively.

### Cardiac mitochondrial oxygen consumption experiments

The ventricle was dissected and permeabilization of cardiac muscle fibers and respirometry were performed to assess mitochondrial respiration as described elsewhere^69,70^. The permeabilized fibers were placed in the respirometry chambers and a substrate-uncoupler-inhibitor titration (SUIT) protocol was performed as previously described^70^ using: (i) pyruvate and malate (5 mM and 0.5 mM respectively) to measure the leak (non-phosphorylating) state for complex I (CI-LEAK); (ii) + ADP (5 mM) to monitor the phosphorylating state for complex I (CI-OXPHOS); (iii) + succinate (10 mM) to assess maximum phosphorylating state with convergent electrons from complex I and complex II (CI + CII-OXPHOS); (iv) + FCCP (titration of 0.25 µM steps) to trigger non-coupled respiration and measure the ETS maximum capacity (CI + CII-ETS); (v) + rotenone (1 µM) + antimycin A (2.5 µM) to inhibit complexes I and III, and measure residual oxygen consumption which was used to correct all the mitochondrial respiration rates; and finally (vi) Ascorbate (2 mM) + TMPD (0.5 mM) were added after raising the oxygen concentration in the chamber to evaluate the maximum capacity of complex IV, which was corrected for auto-oxidation of TMPD. All measurements are presented as means of mass-specific mitochondrial respiration rates (N = 10 for each population at each assay temperature *i.e.* 16 and 23 °C) expressed as pmol O_2_ s^−1^ mg^−1^ of permeabilized fibers ± s.e.m.

### Mitochondrial ratios

With the respiration rates measured above, the P_I_/L_I_ ratio at the level of complex I (CI − OXPHOS/CI-LEAK), as well as the E_I+II_/P_I+II_ ratio (CI + CII-ETS/ CI + CII-OXPHOS) were calculated. The P_I_/L_I_ ratio represents the mitochondrial respiration supporting ATP synthesis to that required to offset the proton leak and is indicative of mitochondrial quality and of mitochondrial coupling^50,71^. The E_I+II_/P_I+II_ ratio is an expression of the limitation of OXPHOS capacity by the phosphorylation system^50^.

### Activity of cardiac antioxidant enzymes and oxidative damages to lipids and proteins

Ventricular tissues were homogenized in 50 mM potassium phosphate buffer complemented with 1 mM EDTA, pH 7.2. The homogenate was divided into four aliquots. Each aliquot was centrifuged either at 10,000×*g* for 15 min, 1600×*g* for 10 min, 13,000 × *g* for 3 min or 1500×*g* for 5 min, for carbonyls, TBARS, CAT and SOD determination, respectively. The resulting supernatants were either frozen at − 80 °C for assessment of oxidative damages to lipids (according to TBARS levels, N = 20) and proteins (carbonyls, N = 20), or directly used for the measurement of SOD and catalase CAT activities (N = 20 for each assay temperature). TBARS and carbonyls were measured using an EnVision Multilabel plate Reader (PerkinElmer, Waltham, MA, USA) set at room temperature. SOD and CAT activities were measured at both 16 and 23 °C using a cuvette UV/VIS spectrophotometer Lambda 11 (PerkinElmer) equipped with a thermostat controlled cell holder and a circulating water bath. All parameters were normalized by total protein content determined using bicinchoninic acid with BSA as standard^72^.

#### Oxidative damages in the heart

TBARS levels were measured using the TBARS assay kit from Cayman Chemical (Ann Harbor, MI, United States). Briefly, the samples were incubated with thiobarbituric acid at high temperature (90–100 °C). The adducts formed by the reaction were determined fluorimetrically at an excitation wavelength of 530 nm and an emission wavelength of 550 nm against a standard. Results are expressed as μmol of TBARS formed per g of tissue ± s.e.m. Carbonylation of proteins was measured with the Protein Carbonyl Colorimetric Assay kit (Cayman Chemical) according to the manufacturer protocol, using the DNPH reaction. The amount of protein-hydrozone produced was quantified spectrophotometrically at 370 nm. The carbonyl content is expressed as nmol of protein carbonyl per mg of proteins ± s.e.m.

#### Antioxidant enzyme activities

Total SOD activity was measured in fresh homogenates at 16 and 23 °C using a Superoxide Dismutase Assay kit from Cayman Chemical (Ann Harbor, MI, United States) following the manufacturer protocol. Briefly, this assay follows the superoxide radicals generated by xanthine oxidase and hypoxanthine using tetrazolium salt for spectrophotometric detection at 450 nm. Total SOD is expressed as means of U mg^−1^ proteins ± s.e.m. where one unit of SOD is defined as the amount of enzyme needed to exhibit 50% dismutation of the superoxide radical. CAT activity was measured as previously described^43^. Briefly, the samples were incubated with 60 mM H_2_O_2_ and the decrease in absorbance corresponding to the decomposition rate of H_2_O_2_ was measured at 240 nm for 4 min. Catalase activity is expressed as mU mg^−1^ proteins ± s.e.m.

### Cardiac mitochondrial gene expression and genotyping

Total RNA was isolated from ventricles of the reference and Biotest fish (N = 10 for each population) using TRIzol reagent (Sigma–Aldrich, St Louis, MO, USA) according to the manufacturer protocol. The 260 nm/280 nm absorbance ratio was used to verify the quality of the RNA in each sample. Total RNA (1 μg) was reverse transcribed using the iScript cDNA synthesis kit (Biorad). Real-time quantitative PCR was performed on a CFX Connect (Biorad, Hercules, CA, USA) by incubating the cDNA with forward and reverse primers and the SsoAdvanced Universal SYBR Green Supermix (Biorad) using the following protocol: denaturation for 2 min at 95 °C, followed by 40 cycles of 15 s at 95 °C and 30 s at 59 °C. Oligonucleotide primers were used to detect the gene expression of *nd4*, *cox1*, *β-actin* and *ef1-α* (Table S1). The relative quantification of gene expression between the two populations was calculated with the 2^−ΔΔCt^ method using both *β-actin* and *ef1-α* as reference genes^73^.

Total DNA was isolated from the ventricles with a Qiagen DNeasy Blood & Tissue Kit (QIAGEN Inc., Valencia, CA, USA). The quality and quantity of DNA, respectively, were assessed by electrophoresis on 1% agarose gels and with a BioDrop µLITE spectrophotometer. Partial sequence amplifications of *cox1*, *cytb* and *16S* were carried out in 50 µl volumes comprising 5.0 µl 10X Taq buffer, 1.0 µl dNTP mix (10 mM), 2.0 µl of each forward and reverse primer [10 µM; PerCox1F 5′-gctggtaccggatgaactgt-3′ and PerCox1R 5′-tggtgagcccacacaataaa-3′for *cox1;* PerCytbF 5′-ccttacatcggcaatgacct-3′ and PerCytbR 5′–ttcctccaattcaggtgagg-3′ for *cytb*; and 16Sar and 16Sbr for *rrnL]*, 0.25 µl Taq DNA Polymerase (5 U/µl; Bio Basic Inc., Markham, ON, Canada), 2 µl of DNA extract (100 ng/μl), and ddH_2_O up to 50 µl. Reactions were performed on a TProfessional Basic Thermocycler with the following PCR amplification conditions: initial denaturation at 95 °C for 2 min, followed by 35 cycles of 95 °C for 20 s, 54 °C for 40 s, and 72 °C for 40 s, followed by a final extension step at 72 °C for 5 min. Resulting PCR products were visualized on 1% agarose gels under UV light with SYBR green dye (Life Technologies), and purified with the Qiagen QIAquick PCR Purification Kit according to the manufacturer protocol. The purified PCR products were sequenced at the Genome Quebec Innovation Centre (McGill University), using the Applied Biosystem’s 3730xl DNA Analyzer technology. Sequences were edited and aligned using MEGA 6 (version6.06)^74^.

### Statistical analysis

All statistical analyses were performed with R software^75^. For all the parameters measured, the data were fitted to a linear model with the body mass as covariate. Normality of residuals was checked, homogeneity of variances was verified using the Levene’s test, and data were transformed when required. Population (Biotest and reference) and Assay Temperature (16 and 23 °C) were included as fixed effects, and their interaction was tested. To analyze mitochondrial respiration rates as well as enzymatic activities of catalase and superoxide dismutase, two-way ANOVAs were performed. For the TBARS levels, protein carbonyl content, as well as for relative gene expression, a one-way ANOVA was performed using Population (Biotest and reference) as fixed factor. In all cases, multiple comparisons were tested with pairwise comparisons of the least-squares means using adjusted P-values (Tukey method) with significance set at P ˂ 0.05.

### Ethical approval

Experimental protocols were approved and performed in agreement with the ethical permits 65–2012 from the animal ethics committee in Gothenburg (Sweden). All methods were carried out in accordance with relevant guidelines and regulations.

## Supplementary information

Supplementary file1

Supplementary file2

## Data Availability

The datasets for this manuscript will be uploaded as part of the supplementary material upon acceptance of the manuscript.
