# Supplementary material for: Adjustments of cardiac mitochondrial phenotype in a warmer thermal habitat is associated with oxidative stress in European perch, Perca fluviatilis
Source: Sci Rep. 2020 Oct 19;10:17697. doi: 10.1038/s41598-020-74788-1 (PMC7572411; doi:10.1038/s41598-020-74788-1)
Supplement: Supplementary file 1 — Supplementary file1 [file 41598_2020_74788_MOESM1_ESM.pdf]

Adjustments of cardiac mitochondrial phenotype in a warmer thermal habitat is associated with oxidative stress in European perch, *Perca fluviatilis*.

Nicolas Pichaud[1,2,3,*], Andreas Ekström[2], Sophie Breton[4], Fredrik Sundström[5], Piotr Rowinski[5], Pierre U. Blier[3], Erik Sandblom[2].

[1] Department of Chemistry and Biochemistry, Université de Moncton, Moncton, NB, Canada, E1 A 3E9

[2] Department of Biological and Environmental Sciences, University of Gothenburg, Gothenburg, Sweden, 405 30

[3] Department of Biology, Université du Québec à Rimouski, Rimouski, QC, Canada, G5L 3A1

[4] Department of Biological Sciences, Université de Montréal, Montréal, QC, Canada, H2V 2S9

[5] Department of Ecology and Genetics, Uppsala University, Uppsala, Sweden, 752 36

* Corresponding author: nicolas.pichaud@umoncton.ca

Supplementary information

Supplementary table

Table S1. Primers used for q RT PCR

Gene		Primer Sequence (5'-3')	Product Size (bp)	Tm (°C)	Accession nº
COI	F	ttctccttcttgcttcctcagg	85	59.5	KC819887
	R	tgcgctaaattcccagcaag			
ND4	F	aacaccctgaggcttttcag	82	58.5	HM050129
	R	atttgccaggcagaaaaggg			
EF1-α	F	atgccgccattgtcaaactg	94	60	KC513785
	R	atgtcacgcacagcaaaacg			
β-actin	F	tatgtgcaaagccggtttcg	123	59.7	EU664997
	R	tcaccaacgtagctgtccttc			
